# Adaptive Immunity, Inflammation, and Cardiovascular Complications in Type 1 and Type 2 Diabetes Mellitus

**DOI:** 10.1155/2013/184258

**Published:** 2013-05-23

**Authors:** Daniela Pedicino, Giovanna Liuzzo, Francesco Trotta, Ada Francesca Giglio, Simona Giubilato, Francesca Martini, Francesco Zaccardi, Giuseppe Scavone, Marco Previtero, Gianluca Massaro, Pio Cialdella, Maria Teresa Cardillo, Dario Pitocco, Giovanni Ghirlanda, Filippo Crea

**Affiliations:** ^1^Institute of Cardiology, Catholic University, Largo A. Gemelli, 8-00168 Rome, Italy; ^2^Diabetes Care Unit, Internal Medicine, Catholic University, Largo A. Gemelli, 8-00168 Rome, Italy

## Abstract

Diabetes mellitus (DM) is a pandemics that affects more than 170 million people worldwide, associated with increased mortality and morbidity due to coronary artery disease (CAD). In type 1 (T1) DM, the main pathogenic mechanism seems to be the destruction of pancreatic **β**-cells mediated by autoreactive T-cells resulting in chronic insulitis, while in type 2 (T2) DM primary insulin resistance, rather than defective insulin production due to **β**-cell destruction, seems to be the triggering alteration. In our study, we investigated the role of systemic inflammation and T-cell subsets in T1- and T2DM and the possible mechanisms underlying the increased cardiovascular risk associated with these diseases.

## 1. Introduction

Diabetes mellitus (DM) is a pandemics that affects more than 170 million people worldwide [1, 2], associated with increased mortality and morbidity due to coronary artery disease (CAD) [3, 4]. Patients with DM have 2- to 4-fold increase in risk of CAD and up to 3-fold increase in mortality and carry the same level of risk for subsequent acute coronary events as nondiabetic patients with previous myocardial infarction (MI). DM also worsens early and late outcomes in acute coronary syndromes (ACS) [5, 6]. Indeed, DM is a prothrombotic condition, associated with inflammation, altered innate immunity, and impaired endothelial function [7, 8]. However, the mechanisms responsible for the higher cardiovascular risk that accompanies DM are multiple and still largely unknown.

In type 1 (T1) DM, the main pathogenic mechanism seems to be the destruction of pancreatic *β*-cells mediated by autoreactive T-cells resulting in chronic insulitis. Thus, the chronic inflammation of pancreatic islets plays a pivotal role in the development of the disease [9]. 

In type 2 (T2) DM, primary insulin resistance, rather than defective insulin production due to *β*-cells destruction, seems to be the triggering alteration. Insulin resistance leads to a perturbation in the lipid homeostasis, cytokines, and adipokines production, resulting in increased systemic inflammation, with higher levels of inflammatory markers such as C-reactive protein (CRP), interleukin (IL)-6, and tumor necrosis factor (TNF)-*α* [7, 10–16].

Inflammation and immunity play also a key role in the pathogenesis of atherosclerosis and its complications. Immune responses are a cornerstone in the mechanisms of endothelial activation, plaque development, and rupture, as demonstrated by the correlations between levels of inflammatory markers or certain lymphocyte population and the risk of occurrence of cardiovascular (CV) events [17–21]. Several studies documented a role for T-cell subset imbalance in the induction of an altered glucose homeostasis both in T1DM and in T2DM [22–42]. Recent experimental models demonstrated that interferon (IFN)-*γ*-producing cells are present in elevated number in adipose tissue of obese mice and have a role in promoting a loss in glucose homeostasis, mostly derived by the induction of visceral adipose tissue inflammation and endothelial dysfunction [24, 25]. Among T lymphocytes, CD4^+^CD28^null^ cells represent a subpopulation producing high levels of IFN-*γ*, showing resistance to apoptosis, and exerting direct cytolytic effects on endothelial cells and proapoptotic effects on smooth muscle cells [26–30]. Our group has previously shown that circulating CD4^+^CD28^null^ T-cell frequency higher than 4% is associated with a worse outcome of ACS. Moreover, for the first time, we specifically demonstrated that in T2DM patients CD4^+^CD28^null^ T-cells are expanded and are associated both with the occurrence of a first CV event and with a higher risk of recurrences after an ACS [31, 32]. However, data on this particular T-cell subset and its implication in diabetes are still lacking; particularly, there are no studies exploring the role of CD4^+^CD28^null^ T-cells in T1DM and its complications.

The role of CD4^+^CD25^+^Foxp3^+^ regulatory T-cells (Tregs) in both T1DM and T2DM has also been investigated. In T2DM, Tregs seem to play a fundamental part in the regulation of body weight, adipocyte hypertrophy, glucose tolerance insulin resistance, and thus in the disease progression [24, 33, 34]. Also, in T1DM, Tregs seem to be involved in the onset and development of the disease, as suggested by studies documenting that alteration of Treg compartment may cause or predispose to autoimmunity [35–37]. The possible mechanism linking Tregs, autoimmunity, and T1DM has been evaluated in recent studies showing that Tregs in the pancreas of NOD mice are prone to apoptosis and fail to control autoimmune responses leading to diabetes [38]. Tregs have also a role in the suppression of the inflammatory and immune responses leading to CV diseases [39, 40]. Recently, a powerful inhibition of atherosclerosis mediated by naturally arising Tregs has been demonstrated in mouse models [41]. Nevertheless, the last decade has seen controversial reports on defects in frequency or function of Tregs in T1DM [42]. 

Aim of the present study is to investigate the role of systemic inflammation and T-cell subsets in T1 and T2DM and the possible mechanisms underlying the increased CV risk, which characterize these conditions.

## 2. Materials and Methods

### 2.1. Study Population

#### 2.1.1. DM Patients

We enrolled 110 patients with a diagnosis of DM defined according to ADA criteria, 55 of them affected by T1DM and 55 patients with T2DM. Exclusion criteria were (1) age > 80 years (*n* = 37); (2) evidence of inflammatory or infectious diseases, malignancies, immunologic, or hematological disorders (*n* = 34); (3) treatment with anti-inflammatory drugs other than low-dose aspirin (*n* = 32).

Clinical features were carefully recorded, including classical cardiovascular risk factors, age at diagnosis of DM, duration of DM, glycosylated haemoglobin A1c (HbA1c), and body mass index (BMI). A complete cardiovascular screening was performed, including a standard 12-lead EKG, a treadmill EKG stress test, an Echo-color Doppler of carotid arteries, the ankle brachial pressure index, and an Echo-color Doppler to exclude lower-limb arterial disease. Microvascular complications, including diabetic nephropathy, neuropathy, and retinopathy, were also assessed. To address the presence of these possible complications, a complete neurological examination performed by certified independent neurologists, a quantitative measurement of urine protein excretion in a sample obtained from 24 h urine collection, and a fundoscopy performed by certified independent ophthalmologists were used.

#### 2.1.2. Controls

As control group, we studied 60 individuals without overt cardiovascular disease and DM, who were screened in our outpatients clinic for cardiovascular prevention. We ascertained traditional cardiovascular risk factors including age, blood pressure, smoking status, family history of MI, and total, LDL, HDL cholesterol and triglycerides levels.

All individuals gave their written informed consent. The Ethics Committee of the catholic University of Rome approved the study.

### 2.2. Anthropometric Measurements

Anthropometric measurements were taken according to standardized procedures after an overnight fasting, with the patient wearing indoor clothes without shoes. Height was measured in centimeters using a stadiometer. Weight was measured with Tanita bioimpedance balance (Tanita International Division, West Dryton, UK). Waist circumference was measured just above the uppermost lateral border of the right ileum using the National Health and Nutrition Examination Survey protocol [43].

### 2.3. Blood Sampling

Venous blood samples were taken at the time of patient enrollment. 

Total and differential white blood cell counts and T-cell subset distribution were analyzed on fresh blood samples. Whole blood samples were also used to assess HbA1c levels, whereas coded serum samples were stored at −70°C and analyzed for high-sensitivity C-reactive protein (hs-CRP) in a single batch at the end of the study by laboratory staff unaware of the clinical data.

### 2.4. T-Cell Analysis

Total and differential white blood cell counts were obtained with a Bayer H*3-hematology analyzer using automated cytochemistry in flow.

T-cell subsets were assessed by flow cytometry. Peripheral blood mononuclear cells (PBMCs) were isolated from heparinized whole blood samples by standard gradient centrifugation over Ficoll-Hypaque (GE Healthcare Bio-Sciences, Piscataway, NJ, USA), washed twice in PBS, then resuspended to a density of 10^5^ cells/mL, stained with the appropriate monoclonal antibodies for cell surface staining, and fixed. When indicated, PBMCs were permeabilized by permeabilization buffer (eBioscience, San Diego, CA, USA) for intracellular staining. Isotype controls were given to enable correct compensation and confirm antibody specificity. In all cases, nonspecific staining with isotype-matched control mAb was <1%; the intra- and interassay variability was <10%. Analyses of stained cells were performed using an FC500 Flow Cytometry System (Beckman Coulter, Fullerton, CA, USA). CXP ACQUISITION and CXP ANALYSIS software packages (Beckman Coulter, Fullerton, CA, USA) were used for data acquisition and analysis, respectively. All T-cell subsets analyzed were expressed as a percentage of the entire population of CD4^+^ T-cells.

#### 2.4.1. Frequency of Different T-Cell Subsets

CD4^+^CD28^null^ T-cell frequency was determined using anti-CD4-fluorescin isothiocyanate- (FITC-) conjugated mAb and anti-CD28-phycoerythrin- Cy5(PC5-) conjugated mAb (both Beckman Coulter, Fullerton, CA, USA). 

Treg cells were defined as CD4^+^Foxp3^+^ T-cells. To this purpose, PBMCs were stained with anti-CD4-FITC mAb. After cell surface staining, cell fixation, and permeabilization, cells were stained with the intracellular PE-conjugated anti-Foxp3 mAB (eBioscience, San Diego, CA, USA) according to the manufacturer's instructions.

A cutoff value ≥4% was chosen to define patients with high frequency of CD4^+^CD28^null^ T-cells, because 4% represents the 90th percentile of distribution in healthy individuals. This cutoff was also validated in our control group (median = 1.5%; 90th percentile = 4.0%).

A cutoff value ≤5% was chosen to define patients with low frequency of Tregs, because 5% represents the 10th percentile of distribution in our control group (median = 7.8%; 10th percentile = 5.0%).

The CD4^+^CD28^null^/Treg balance was evaluated by the ratio of CD4^+^CD28^null^ T-cells to CD4^+^Foxp3^+^ T-cells; a cutoff value ≥1.1 was chosen to define an alteration of the balance, because this value represents the 99th percentile of distribution in our control group (median = 0.21; 99th percentile = 1.1%).

### 2.5. Measurements of hs-CRP and HbA1**c**

We measured hs-CRP concentrations using an ultrasensitive nephelometric method (Siemens HealthCare Diagnostic BN System, Deerfield, IL, USA). The working range of the assay was 0.175 to 1100 mg/L, and the coefficient of variation was <5%. The median normal value for hs-CRP was 0.8 mg/L, with 90% of normal values <3 mg/L.

HbA1c was assessed by high-performance liquid chromatography, using Diamat BioRad (BioRad, Milan, Italy). The HbA1c reference range was 4.3–5.9%.

### 2.6. Statistical Analysis

Because CD4^+^CD28^null^ and Treg frequencies and hs-CRP values did not follow a normal distribution, according to Kolmogorov-Smirnov, data were expressed as median and range, and nonparametric tests were used: the Kruskal-Wallis test with the Dunn's multiple pairwise comparison for comparisons among groups and the Spearman's rank test for correlations. The remaining continuous variables were expressed as mean ± SD and were compared using 1-way ANOVA for repeated measures, with the Bonferroni correction for multiple pairwise comparisons. Proportions were compared using the chi-square test. 

A two-tailed *P* value <0.05 was considered statistically significant. Statistical analysis was performed with SPSS 18.0 software (SPSS Inc., Chicago, Illinois, USA).

## 3. Results

Characteristics of study population are reported in Table 1. 

As compared with both T2DM patients and controls, T1DM patients were younger (*P* < 0.001 for both comparisons), and exhibited higher rate of hypercholesterolemia (*P* < 0.05 for both comparisons). As compared with T2DM, T1DM patients had a longer disease duration (*P* = 0.020), were more often treated with insulin (*P* < 0.001), and had higher levels of total cholesterol (*P* = 0.045) and HDL cholesterol (*P* < 0.001). They also had higher levels of HDL cholesterol than controls (*P* < 0.001).

T2DM patients exhibited higher rate of family history of IHD (*P* = 0.001 versus controls). As compared with T1DM, T2DM patients were more often treated with oral antidiabetic drugs (*P* < 0.001) and had higher BMI and waist circumference (*P* < 0.001 for both comparisons), higher triglycerides levels (*P* = 0.015), and higher rate of macrovascular complications (*P* = 0.005). 

### 3.1. Frequencies of T-Cell Subsets

No differences were found in total T-cell and CD4^+^ T-cell counts among groups (Table 1). 

**Figure 1:**
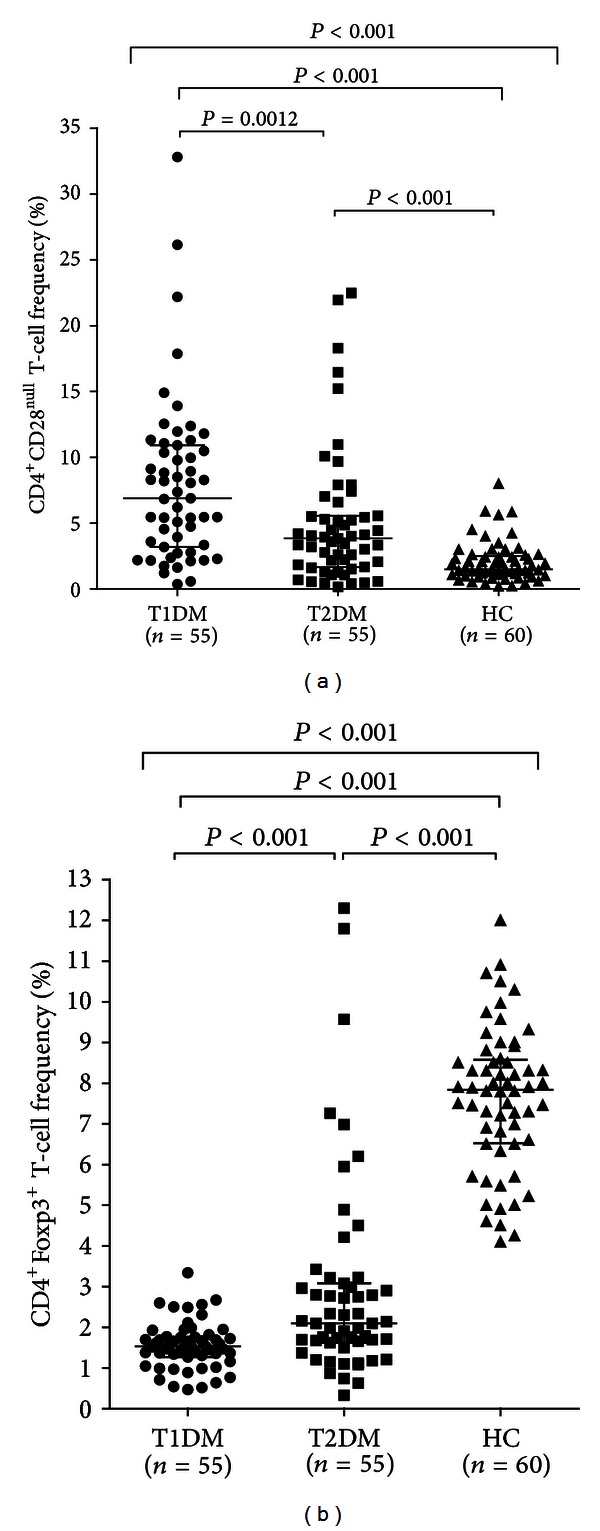
Frequencies of T-cell subsets in different groups. T1DM and T2DM groups were defined according to ADA criteria, while controls were individuals without overt cardiovascular disease and DM. Frequencies of T-cells were determined by two-color flow cytometry. Data are presented as single data points. (a) CD4^+^CD28^null^ T-cell frequencies in different groups. CD4^+^CD28^null^ T-cell frequency was significantly higher in T1DM than in other groups, and it was higher in T2DM than in controls. (b) CD4^+^FoxP3^+^ T-cell frequencies in different groups. CD4^+^FoxP3^+^ T-cell frequency was significantly lower in T1DM than in other groups, and it was lower in T2DM than in controls.

CD4^+^CD28^null^ T-cell frequency was significantly higher in T1DM than in T2DM and in controls (*P* = 0.001 and *P* < 0.001, resp.) and it was higher in T2DM than in controls (*P* < 0.001) (Table 1 and Figure 1(a)). 

In sharp contrast, Treg frequency was significantly lower in T1DM than in T2DM and in controls (*P* < 0.001 for both comparisons), and it was lower in T2DM than in controls (*P* < 0.001) (Table 1 and Figure 1(b)). 

### 3.2. Imbalance between Effector and Regulatory T-Cells in T1DM Patients

**Figure 2:**
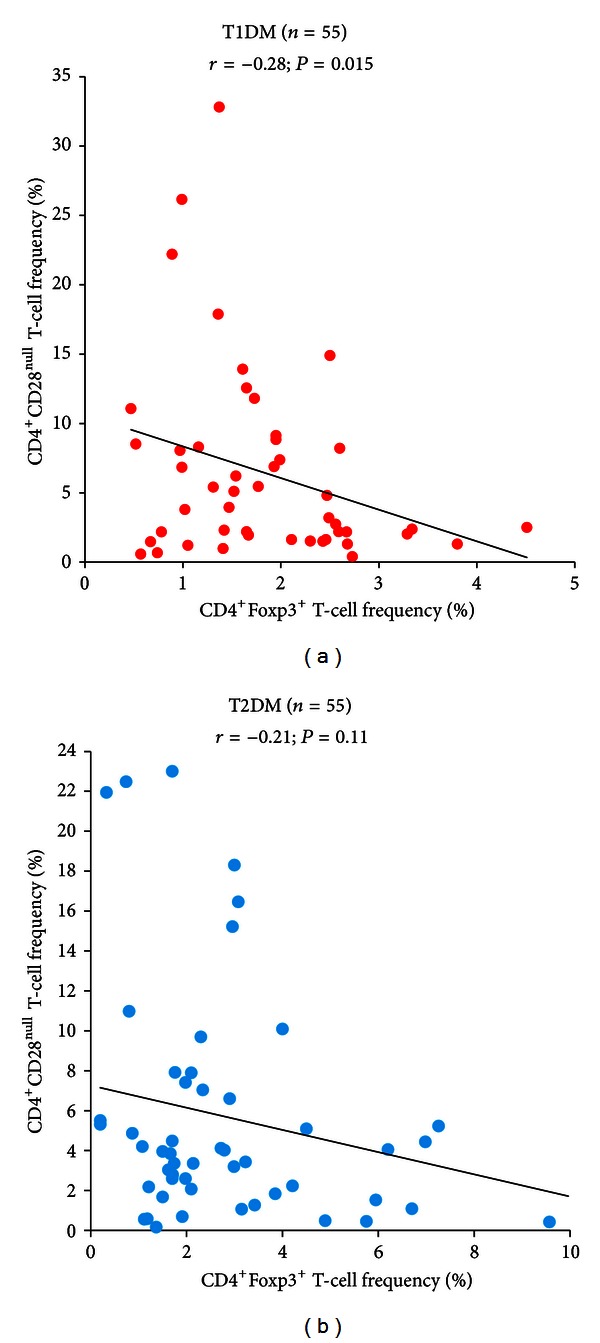
Correlation between CD4^+^CD28^null^ T-cell and CD4^+^FoxP3^+^ T-cell frequencies in T1DM and T2DM patients. Data are presented as single data points. (a) In T1DM patients, a significant negative correlation was found between CD4^+^CD28^null^ T-cell and CD4^+^FoxP3^+^ T-cell frequencies. (b) In T2DM patients, no correlation was found between CD4^+^CD28^null^ T-cell and CD4^+^FoxP3^+^ T-cell frequencies.

In T1DM, a statistically significant negative correlation was detected between CD4^+^CD28^null^ T-cell frequency and Tregs (*R* = −0.28; *P* = 0.015), likely suggesting the preferential association of increased CD4^+^CD28^null^ T-cell immune response with impaired Treg function in single T1DM patients (Figure 2(a)). No correlation was observed in T2DM (Figure 2(b)). 

**Figure 3:**
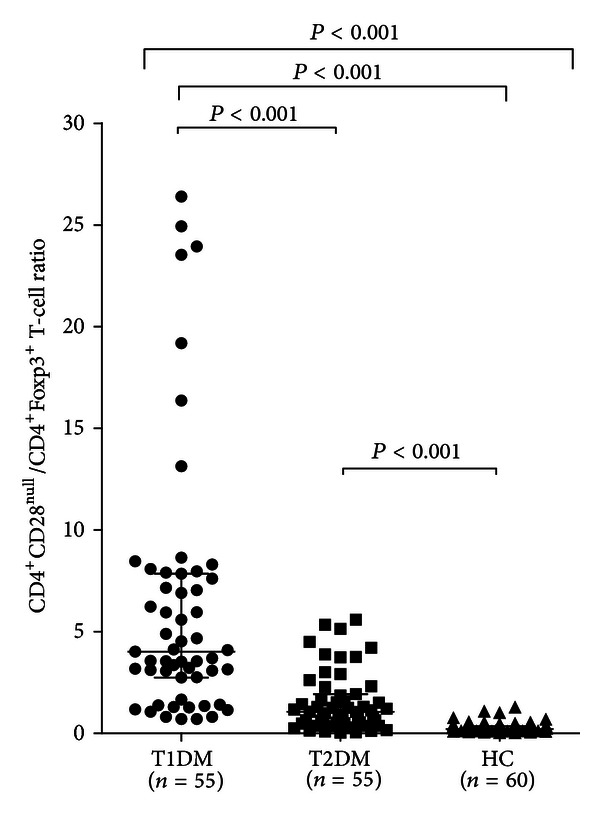
CD4^+^CD28^null^/CD4^+^FoxP3^+^ percentage ratio in different groups. T1DM, T2DM, and control groups were defined as in Figure 1. Data are presented as single data points. A significantly higher CD4^+^CD28^null^/CD4^+^FoxP3^+^ ratio was detected in T1DM patients than in T2DM and in controls. CD4^+^CD28^null^/CD4^+^FoxP3^+^ ratio was also higher in T2DM patients than in controls.

The significance of increased CD4^+^CD28^null^ T-cell frequency and decreased Treg cells was further explored by calculating the CD4^+^CD28^null^/Treg cell percentage ratio in each subject. There was a strikingly higher CD4^+^CD28^null^/Treg ratio in T1DM patients than in T2DM patients and in controls (*P* < 0.001 for all comparisons), and a higher ratio in T2DM patients than in controls (*P* < 0.001) (Table 1 and Figure 3). 

### 3.3. Systemic Inflammation

**Figure 4:**
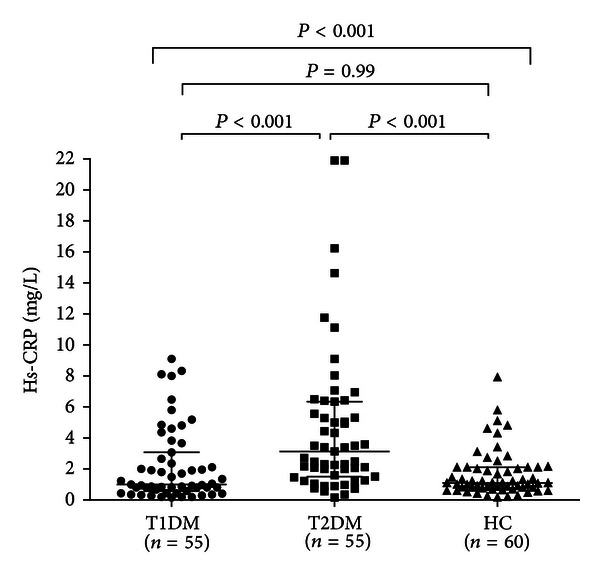
Serum hs-CRP levels in different groups. Data are presented as single data points. Serum hs-CRP levels were significantly higher in T2DM than in the other groups. No difference was found between T1DM patients and controls.

We have also studied low-grade systemic inflammation assessing the serum levels of hs-CRP. Hs-CRP was significantly higher in T2DM than in the other groups (*P* < 0.001 for all comparisons). Of note, T1DM patients had similar hs-CRP levels than controls (*P* = 0.99) (Table 1, Figure 4). 

### 3.4. T-Cell Frequencies and hs-CRP Levels according to Metabolic Factors, Disease Duration, and Complications

In T1DM patients, no correlations were observed between CD4^+^CD28^null^ T-cell and/or Treg frequency and metabolic factors, disease duration, and complications.

**Figure 5:**
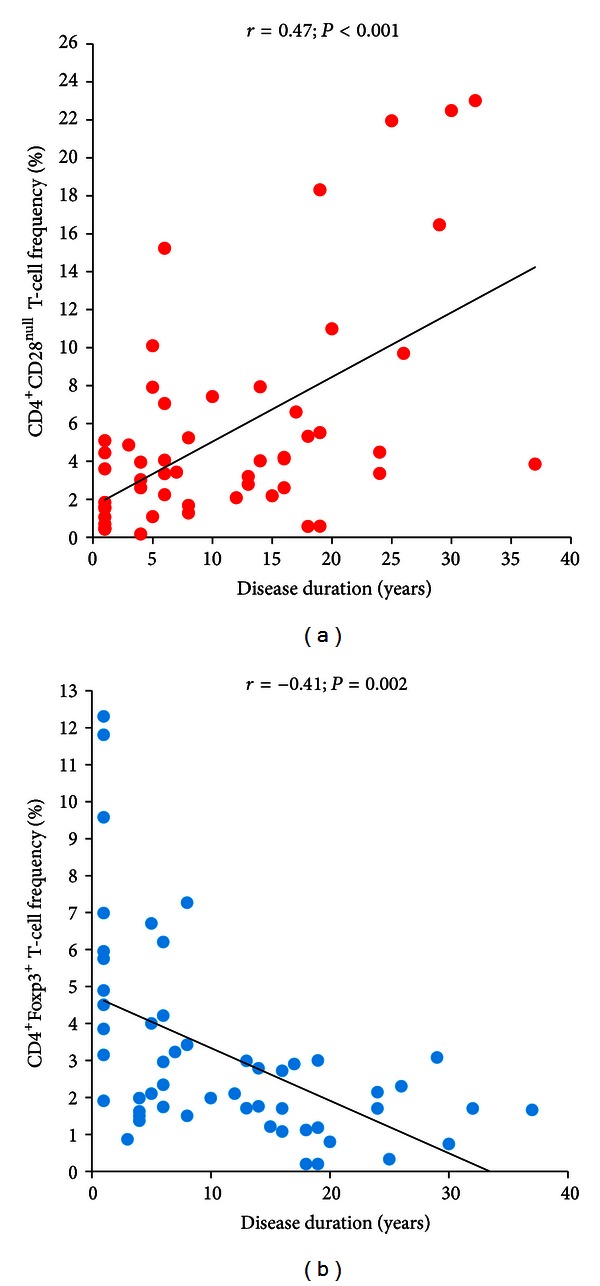
Correlation between T-cell subsets and disease duration in T2DM patients. T2DM patients were defined as in Figure 1. Data are presented as single data points. (a) CD4^+^CD28^null^ T-cell frequency positively correlates with disease duration in T2DM patients. (b) CD4^+^FoxP3^+^ T-cell negatively correlates with disease duration in T2DM patients.

**Figure 6:**
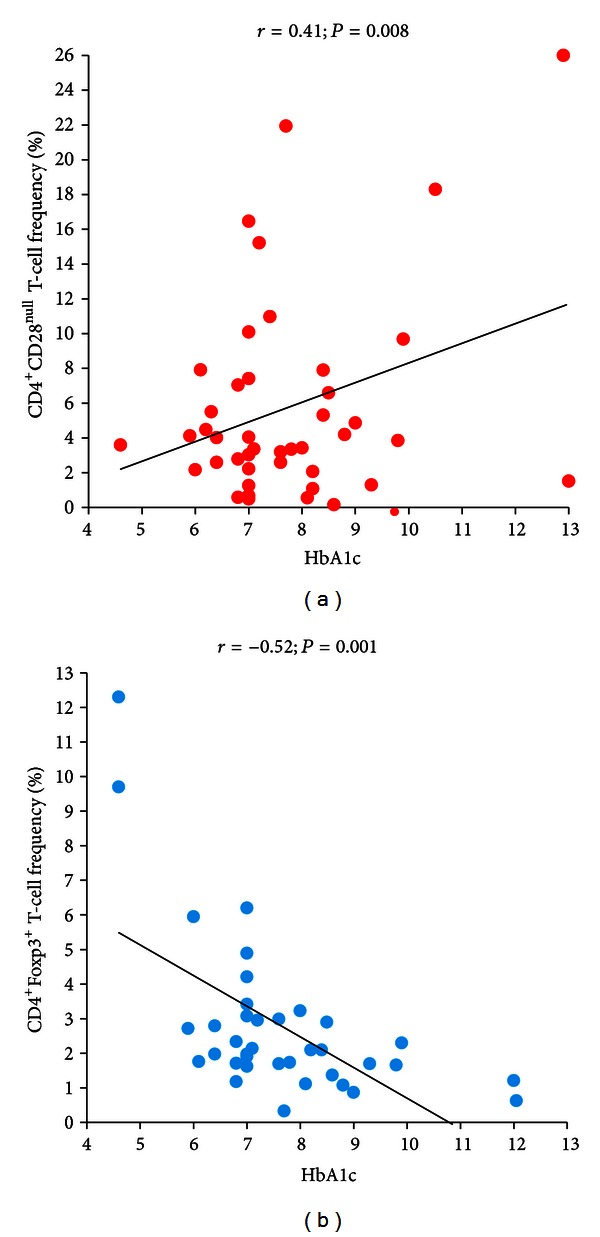
Correlation between T-cell subsets and serum HbA1c levels in T2DM patients. T2DM patients were defined as in Figure 1. Data are presented as single data points. (a) CD4^+^CD28^null^ T-cell frequency positively correlates with HbA1c in T2DM patients. (b) CD4^+^FoxP3^+^ T-cell negatively correlates with HbA1c in T2DM patients.

**Figure 7:**
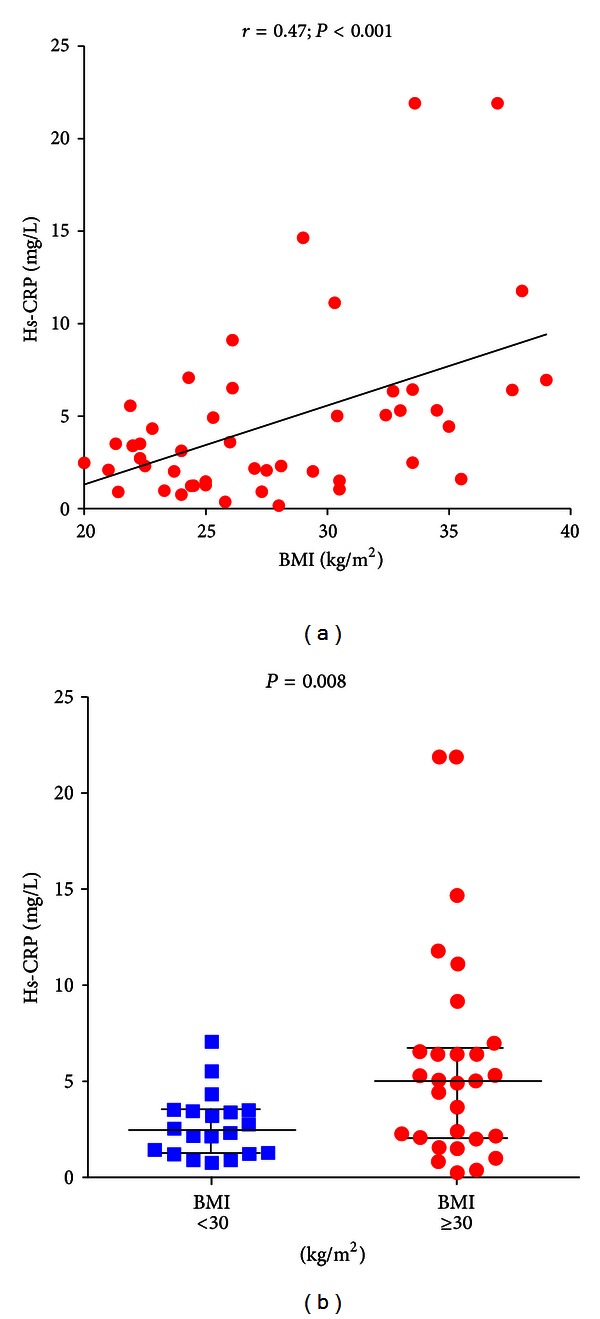
Correlation between serum hs-CRP levels and BMI in T2DM patients. T2DM patients were defined as in Figure 1. Data are presented as single data points. (a) Positive correlation was statistically found between serum hs-CRP levels and BMI in T2DM patients. (b) Obese T2DM patients (BMI ≥ 30 kg/m^2^) showed higher levels of serum hs-CRP than nonobese (BMI < 30 kg/m^2^) T2DM patients.

In T2DM patients, CD4^+^CD28^null^ T-cell frequency was positively correlated (*r* = 0.47; *P* < 0.001), and Treg frequency was negatively correlated (*r* = −0.41; *P* = 0.002) with disease duration (Figures 5(a) and 5(b)); moreover, CD4^+^CD28^null^ T-cell frequency was positively correlated (*r* = 0.41; *P* = 0.008), and Treg frequency was negatively correlated (*r* = −0.52; *P* = 0.001) with HbA1c (Figures 6(a) and 6(b)); finally, a statistically significant correlation was found between hs-CRP levels and BMI (*r* = 0.47; *P* < 0.001) (Figure 7).

To investigate the potential relationship between CD4^+^ T-cell subset alteration with the types of diabetic complications, we compared the levels of CD4^+^CD28^null^ T-cells and Tregs in T1DM and T2DM patients with or without different types of complications. No differences were observed among T1DM patients with or without micro- or macrovascular complications. CD4^+^CD28^null^ T-cells and Treg were also similar in T2DM patients with or without micro- or macrovascular complications, while hs-CRP levels were significantly higher in T2DM patients with macrovascular complications (median 4.3, range 0.5–21.9 versus median 2.0, range 0.5–14.6; *P* = 0.047). 

These data suggest that the changes of CD4^+^ T-cell subsets in T1DM patients may happen before the occurrence of complications and at the early stage of disease. In contrast, in T2DM patients, CD4^+^ T-cell subset alteration might be the consequence of disease duration and metabolic control.

## 4. Discussion

DM and CAD are two pathological conditions closely related to each other; as demonstrated by the fact that, in the evaluation of cardiovascular risk, DM is considered as an equivalent of established CAD [3, 4]. Atherothrombosis is the leading cause of death among diabetic patients, accounting for about 80% of mortality in this population, which comprehends more than 150 million people worldwide; therefore, the burden of DM-related CAD constitutes a major health problem [3, 4].

Several mechanisms could be involved in the pathogenesis of diabetes-related vascular complications, including oxidative stress, vascular damage due to advanced end glycation products (AGE), and glucose-induced inflammation [7, 8, 13, 14]. 

It is well established that both T1DM and T2DM are associated with a systemic inflammatory state [44–46]. The main features of inflammation, however, could be different between T1DM and T2DM, due to their different pathogenesis. In fact, T1DM onset is related to pancreatic *β*-cells destruction by autoreactive T-cells and subsequent insulin deficiency leading to the metabolic alterations of the disease [9, 45], while the pathogenesis of T2DM involves primarily insulin resistance, rather than absolute insulin deficiency, deriving from metabolic alterations in insulin-responsive tissues and resulting in a complex alteration of lipid and glucose homeostasis [17, 18]. These perturbations lead to an imbalance in cytokines and adipokines production resulting in increased systemic inflammation [13, 14]. Conversely, systemic inflammation can itself predispose to the onset of insulin resistance and consequently T2DM, as shown by several studies demonstrating that high levels of inflammation markers (including CRP, fibrinogen, IL-6, and plasminogen activator inhibitor PAI-1) could predict the risk of developing T2DM [7, 8, 11, 12]. CRP is now recognized as a sensitive and useful, although nonspecific, marker of cardiovascular risk [16]. In our work, we found higher levels of hs-CRP in T2DM patients, with values of hs-CRP approximately similar in T1DM and in controls. Moreover, we observed higher levels of hs-CRP in those T2DM patients with overt macrovascular complications. Recent studies highlighted the correlation between high levels of CRP and IL-1*β* in diabetic patients. A statistically significant reduction in CRP, IL-6, and other inflammatory biomarkers was observed after the administration of anti-IL-1*β* antibodies in T2DM patients, suggesting a prominent role of this cytokine in the induction of the chronic inflammatory state characterizing the disease [47, 48] and its possible involvement in the subsequent alteration of the adaptive immune response [49].

The role of the adaptive immune system has also been investigated in the pathogenesis of T1DM and T2DM. Particularly, a disregulation of T-cell compartment seems to be a pivotal feature of both the diseases.

CD4^+^CD28^null^ T-cells, extremely unusual in healthy adults, are expanded in chronic inflammatory disorders such as rheumatoid arthritis. These T-cells have a prominent Th1 phenotype, producing high levels of IFN-*γ*, are resistant to apoptosis, and represent a marker of senescence of the immune system [26, 27]. In the atherosclerotic plaque microenvironment, CD4^+^CD28^null^ T-cells have direct cytolytic effects on endothelial cells and proapoptotic effects on smooth muscle cells; thus, they might contribute to plaque instability [29, 30]. In ACS, CD4^+^CD28^null^ T-cell frequency has been associated with the recurrence of acute coronary events [31]. In DM patients, CD4^+^CD28^null^ T-cells are expanded and are associated with poor glycemic control, with the occurrence of a first cardiovascular event and with a worse outcome after an ACS [32].

At the other extreme, CD4^+^CD25^+^Foxp3^+^ regulatory T-cells (Tregs) have a key role in the suppression of pathological inflammatory and immune responses. Their frequency is reduced in several autoimmune diseases and also in ACS [40, 50–52]. Recently, we have observed that a large subset of ACS patients presents a unique adaptive immunity system signature, associated to a worse outcome and characterized by inadequate Treg response to effector T-cell expansion [52].

Although CD4^+^CD28^null^ T-cells have been implicated in several immunological disorders, no previous studies have assessed the role of this T-cell subset in T1DM. More importantly, in the present study, we have systematically explored the balance between aggressive effector T cells/regulatory T cells by comparing patients with T1DM and T2DM. An altered immune balance has been found in both T1DM and T2DM, although with substantial differences. 

In the present study, T1DM patients were characterized by higher CD4^+^CD28^null^ T-cell frequency and lower Treg frequency, by a strikingly higher CD4^+^CD28^null^/Treg ratio, and by a negative correlation between CD4^+^CD28^null^ T-cells and Tregs. These findings suggest the preferential association of increased CD4^+^CD28^null^ T-cell immune response with impaired Treg function in single T1DM patients. All together, our data suggest that, in the altered response of adaptive immunity involved in the pathogenesis of T1DM, a role might be reserved to special subset of T-cells, which could represent both the triggering factor and the continuing stimulus sustaining the disease. On the other hand, in T2DM, an imbalance in the adaptive immunity seems to appear later in the course of the disease as a consequence of a chronic systemic inflammatory state that is involved both in the pathogenesis of the disease and in its macrovascular complications. In fact, an altered adaptive immunity is also present in T2DM patients with CD4^+^CD28^null^ T-cell frequency higher and Treg frequency lower than in controls, but it seems to be strictly related to disease duration and glycemic control rather than being a mechanism involved in the early phase of the disease. In our previous study, we demonstrated that the expansion of CD4^+^CD28^null^ T-cells in T2DM patients is independently associated with HbA1c levels and not with fasting glucose [32]. This suggests that the development of unusual T-cell subsets might be influenced by persistent poor glycemic control and might be presumably related to the presence of high levels of AGEs, known to be implicated in the onset of DM vascular complications [14]. 

## 5. Conclusions

Our study demonstrates that both T1DM and T2DM are associated with an impaired T-cell balance, characterized by CD4^+^CD28^null^ T-cell expansion and CD4^+^CD25^+^Foxp3^+^ regulatory T-cell reduction. However, the meaning of these imbalances might be different between the two diseases. In fact, the altered adaptive immunity found in T1DM seems to be both the triggering factor and the continuing stimulus sustaining the disease, while in T2DM the same disregulation seems to appear later in the course of the disease, as consequence of a persistent poor glycemic control and of a chronic systemic inflammatory state which is associated with overt macrovascular complications.

Our results pave the way to a novel explanation for the possible role of adaptive immune system in the development of T1DM and T2DM, which could also have important implications in the prevention and treatment of diabetes and its cardiovascular complications. Interestingly, recent trials showed that IL-1*β* neutralising antibodies improve glycemic control and reduce inflammation in patients with T2DM [47, 48]. Further studies are needed to ascertain whether the defective number of Tregs is part of the causation leading to T1DM or an epiphenomenon. In the meantime, a number of immunomodulatory intervention trials have now been conducted in patients at risk for or with recent onset T1DM, often with the goal of restoring immune tolerance by inducing Tregs [53–55].

## Figures and Tables

**Table 1:** Clinical characteristics of study population.

	T1DM	T2DM	Controls	*P* value
Number of patients	55	55	60	
Sex (M/F)	37/18	36/19	34/26	0.118
Age (mean ± SD)	45.2 ± 14	62 ± 10	55.7 ± 11.8	0.037^a^
Risk factors				
Hypercholesterolemia, *n* (%)	31 (56%)	20 (36%)	17 (28%)	0.048^b^
Hypertension, *n* (%)	26 (47%)	37 (67%)	48 (80%)	0.015^c^
Smoke, *n* (%)	22 (40%)	19 (35%)	26 (43%)	0.63
Family history of IHD, *n* (%)	26 (47%)	37 (67%)	18 (30%)	0.002^d^
Medications				
Oral antidiabetic drugs, *n* (%)	8 (15%)	44 (80%)	NA	<0.001
Insulin, *n* (%)	54 (98%)	15 (27%)	NA	<0.001
Statins, *n* (%)	25 (45%)	24 (44%)	7 (12%)	<0.001^e^
Disease complications				
Microvascular complications (nephropathy, neuropathy, and retinopathy)	25 (45%)	28 (51%)	NA	0.33
Macrovascular complications (CAD, PVD, and cerebrovascular diseases)	18 (33%)	37 (67%)	NA	0.005
Antropometric parameters (mean ± SD)				
BMI (kg/m^2^)	24.4 ± 3.5	27.8 ± 5.2	25.9 ± 3.8	0.003^f^
Waist circumference (cm)	81 ± 10	93 ± 12.6	79 ± 7	0.002^f^
HbA1c (%)	7.4 ± 0.4	7.6 ± 1.4	NA	0.418
Mean duration of DM (years)	18.5 ± 10.4	13.5 ± 8.9	NA	0.020
Laboratory assay (mean ± SD)				
Total cholesterol (mg/dL)	196.5 ± 30	178.5 ± 49.3	183.5 ± 43.5	0.018^g^
LDL (mg/dL)	100.9 ± 44.7	104.6 ± 37.8	112.2 ± 40.1	0.65
HDL (mg/dL)	62 ± 13.7	49.6 ± 11	50.3 ± 15.6	<0.001^a^
Triglycerides (mg/dL)	99.2 ± 38.4	126.6 ± 61.3	111.1 ± 60.7	0.029^h^
Lymphocyte count (10^9^/L)	1.5 ± 0.5	1.6 ± 0.5	1.9 ± 0.6	0.4
Total CD4^+^ T-cell frequency (%)	50.3 ± 20.2	50.5 ± 19.7	50.4 ± 23.6	0.4
hs-CRP (mg/L), median (range)	1.0 (0.2–9.1)	3.4 (0.2–21.9)	1.1 (0.2–7.9)	<0.001^i^
Frequency of different T-cell subsets				
Expressed as percentage of the entire CD4^+^ T-cell population, median (range)				
CD4^+^CD28^null^ T-cell (%)	6.9 (0.4–32.8)	3.6 (0.2–22.5)	1.5 (0.2–8.0)	<0.001^j^
CD4^+^Foxp3^+^ T-cell (Treg) (%)	1.5 (0.5–3.3)	2.1 (0.3–12.3)	7.8 (4.1–12.0)	<0.001^k^
CD4^+^CD28^null^/Treg ratio	4.3 (0.7–26.4)	1.3 (0.1–66.5)	0.2 (0.1–1.3)	<0.001^l^

DM: diabetes mellitus; IHD: ischemic heart disease; CAD: coronary artery disease; PVD: peripheral vascular disease; BMI: body mass index; HbA1c: glycosylated haemoglobin A1c; hs-CRP: high-sensitivity C-reactive protein.

^a^ *P* < 0.001 T1DM versus T2DM and controls.

^b^ *P* < 0.05 T1DM versus T2DM and controls.

^c^ *P* < 0.001 controls versus T1DM.

^d^ *P* < 0.001 T2DM versus controls.

^e^ *P* < 0.05 T1DM and T2DM versus controls.

^f^ *P* < 0.001 T2DM versus T1DM and controls.

^g^ *P* < 0.05 T1DM versus T2DM.

^h^ *P* < 0.05 T2DM versus T1DM.

^i^ *P* < 0.001 T2DM versus T1DM and controls.

^j^ *P* < 0.001 T1DM (higher frequency) versus T2DM and controls; also, *P* < 0.001 T2DM versus controls.

^k^ *P* < 0.001 T1DM (lower frequency) versus T2DM and controls; also, *P* < 0.001 T2DM versus controls.

^l^ *P* < 0.001 T1DM versus T2DM and controls; also, *P* < 0.001 T2DM versus controls.
